# Reduced inflammatory response in cigarette smoke exposed Mrp1/Mdr1a/1b deficient mice

**DOI:** 10.1186/1465-9921-8-49

**Published:** 2007-07-07

**Authors:** Margaretha van der Deen, Wim Timens, Hetty Timmer-Bosscha, Barry W van der Strate, Rik J Scheper, Dirkje S Postma, Elisabeth G de Vries, Huib A Kerstjens

**Affiliations:** 1 Medical Oncology Department, University Medical Center Groningen and University of Groningen, The Netherlands; 2 Pathology Department, University Medical Center Groningen and University of Groningen, The Netherlands; 3 Pathology Department, VU University Medical Center, Amsterdam, The Netherlands; 4 Pulmonology Department, University Medical Center Groningen and University of Groningen, The Netherlands

## Abstract

**Background:**

Tobacco smoke is the principal risk factor for chronic obstructive pulmonary disease (COPD), though the mechanisms of its toxicity are still unclear. The ABC transporters multidrug resistance-associated protein 1 (MRP1) and P-glycoprotein (P-gp/MDR1) extrude a wide variety of toxic substances across cellular membranes and are highly expressed in bronchial epithelium. Their impaired function may contribute to COPD development by diminished detoxification of noxious compounds in cigarette smoke.

**Methods:**

We examined whether triple knock-out (TKO) mice lacking the genes for *Mrp1 *and *Mdr1a/1b *are more susceptible to develop COPD features than their wild-type (WT) littermates. TKO and WT mice (six per group) were exposed to 2 cigarettes twice daily by nose-only exposure or room air for 6 months. Inflammatory infiltrates were analyzed in lung sections, cytokines and chemokines in whole lung homogenates, emphysema by mean linear intercept. Multiple linear regression analysis with an interaction term was used to establish the statistical significances of differences.

**Results:**

TKO mice had lower levels of interleukin (IL)-7, KC (mouse IL-8), IL-12p70, IL-17, TNF-alpha, G-CSF, GM-CSF and MIP-1-alpha than WT mice independent of smoke exposure (*P *< 0.05). IL-1-alpha, IL-6, IL-8, IL-13, IL-17, TNF-alpha, G-CSF, GM-CSF and MCP-1 increased after smoke exposure in both groups, but the increase in IL-8 was lower in TKO than WT mice (*P *< 0.05) with a same trend for G-CSF (*P *< 0.10). Smoke-induced increase in pulmonary inflammatory cells in WT mice was almost absent in TKO mice. The mean linear intercept was not different between groups.

**Conclusion:**

*Mrp1/Mdr1a/1b *knock-out mice have a reduced inflammatory response to cigarette smoke. In addition, the expression levels of several cytokines and chemokines were also lower in lungs of *Mrp1/Mdr1a/1b *knock-out mice independent of smoke exposure. Further studies are required to determine whether dysfunction of MRP1 and/or P-gp contribute to the pathogenesis of COPD.

## Background

Tobacco smoke generates oxidative stress in the lungs and is the principal risk factor for the development of chronic obstructive pulmonary disease (COPD). Main features of COPD are airway inflammation and destruction of alveolar tissue. Little is known about detoxification and elimination processes of noxious substances present in cigarette smoke. Proteins of the ATP-binding cassette (ABC) superfamily such as multidrug resistance-associated protein 1 (MRP1) and P-glycoprotein (P-gp, encoded by the *MDR1 *gene) protect against oxidative stress, chemotherapeutic drugs and xenobiotics [1,2] by transporting a wide variety of toxic substances across cellular membranes. MRP1 and P-gp are highly expressed in human and mouse lung and are mainly located at the basolateral and apical side of bronchial epithelium respectively [3,4]. So far, the function of these ABC transporters in the lung is unknown [5]. They possibly detoxify carcinogenic compounds and other noxious gasses and particles present in tobacco smoke [6]. Thus a defective function may play a role in the pathogenesis of lung cancer and COPD.

*Mrp1 *(-/-) and *Mdr1a/1b *(-/-) mice (in contrast to humans, rodents have two genes for P-gp, called *Mdr1a *and *Mdr1b*), are healthy and fertile under normal conditions [7,8]. Mice that lack *Mrp1 *and both *Mdr1a/1b genes*, from here on called triple knock-out (TKO) mice, seem physiologically normal as well [9]. However, all these knock-out mice are highly sensitive to several chemotherapeutic drugs. In addition,*Mrp1 *(-/-) mice display elevated glutathione levels in tissues that normally have a high MRP1 expression, e.g. in the lungs [7], possibly as a compensation mechanism for the elevated pulmonary oxidative stress. Furthermore, these mice have an impaired inflammatory response [10] that is probably due to lower leukotriene C4 (LTC4, a proinflammatory mediator) excretion by macrophages or granulocytes since LTC4 is a physiologic substrate for MRP1 [11].

We detected lower MRP1 expression in bronchial epithelium of COPD patients compared to healthy ex-smokers [12]. In the present study, we investigated whether TKO mice have a different susceptibility to develop cigarette smoke-induced features of COPD compared to their wild-type (WT) littermates.

## Methods

### Mice

WT male FVB mice were obtained from Harlan (Zeist, the Netherlands). Male FVB *Mrp1/Mdr1a/1b *TKO mice were kindly provided by Drs AH Schinkel and P Borst, the Netherlands Cancer Institute, Amsterdam [13]. Mice were held at the central animal facility of the University of Groningen. The animals received standard rodent food (Hope Farms, Woerden, The Netherlands) and water *ad libitum*. Experiments were approved by the local committee on animal experimentation, and were performed under strict governmental and international guidelines.

### Smoke exposure

Mainstream smoke of research cigarettes (type 2R1, University of Kentucky, KY) was administered by nose-only exposure [14]. The smoke exposure system of the University of Kentucky was used and the system was set up according to instructions of the manufacturer. Six mice per experimental group were exposed to 2 cigarettes per session, 10 puffs per cigarette, twice daily for 5 days per week. Sham control mice were room air-exposed in a separate animal exposure subunit under similar circumstances.

### Preparation of lungs

After 6 months smoke or air exposure, the mice were anesthetized with a mixture of isoflurane, N_2_O, and oxygen, and the trachea was cannulated. Subsequently, the mice were exsanguinated via the abdominal aorta. The right lung was ligated and lung lobes were snap-frozen and stored at -80°C. The left lung was removed, inflated and fixed with formalin with a constant pressure of 25 cm H_2_O for 24 h. Subsequently, the lung was dissected from the trachea and embedded in paraffin for immunohistochemical evaluation and morphometrical analysis [15].

### Morphometrical measurements of emphysema

Three-micron paraffin sections were cut and stained with hematoxylin and eosin (H&E) according to standard methods. Approximately 25 photomicroscopic images per tissue section were prepared at 2.5 × 20 magnifications using a standardized sequence of image capturing. Images with large vessels, conducting airways or pleura occupying 25% or more of the total image were not used for the mean linear intercept (Lmi) analysis which was assessed as a measure of alveolar airspace enlargement by two independent individuals in a blinded manner [15,16]. Mean values per mouse were used for statistical analysis.

### Cytokine and chemokine analysis in lung homogenates

One part of frozen lung tissue (17 to 30 mg) of each mouse was homogenized in buffer (50 mM Tris-HCl, 150 mM NaCl, 0.002% Tween-20, pH = 7.5) in 10% w/v for 1 to 2 min and subsequently centrifuged for 10 min, 12,000 g at 4°C. Supernatants were stored at -80°C until analysis. Cytokines and chemokines that might play a role in cellular defense mechanisms against cigarette smoke or smoke-induced cellular damage were measured by means of a multiplex Luminex ELISA system (Lincoplex Systems, St Charles, MO). These included: interleukin (IL)-1α, IL-1β, IL-2, IL-4, IL-5, IL-6, IL-7, keratinocyte chemoattractant (KC, murine homologue of IL-8), IL-9, IL-10, IL-12p70, IL-13, IL-15, IL-17, tumor necrosis factor-alpha (TNFα), interferon-gamma (IFNγ), granulocyte-colony stimulating factor (G-CSF), granulocyte-macrophage-colony stimulating factor (GM-CSF), IFN-inducible protein-10 (IP-10), MCP-1 (monocyte chemoattractant protein-1), macrophage inflammatory protein 1-alpha (MIP1α) and regulated upon activation, normal T-cell expressed and secreted (RANTES). The concentrations of cytokines and chemokines were expressed as pg/g total lung tissue.

### Immunohistochemical analysis of infiltrates in lung tissue

The presence of inflammatory cells in paraffin embedded lung sections (3 μm) was evaluated with a semi-quantitative score. The total number of infiltrates in three different sections of each lung were counted. The infiltrates were mainly clustered together in groups, not as single cells. Clusters of 25 cells or more were considered positive. Of every mouse lung, the infiltrates in three sections were averaged and the average per experimental group (4–6 mice) was calculated. All sections were cut in a similar way, i.e. in the same plane and 250 micrometer from the start of sectioning of the tissue. In this way, the counting area was similar in all sections.

Specific monoclonal antibodies anti-Neutrophil (Cedarlane, Sanbio, Uden, The Netherlands), MAC-3 and B220 (both from BD Pharmingen, San Jose, CA) were used to detect the presence of neutrophils, macrophages, and B-cells respectively. Detection of these antibodies was performed using biotin-labeled rabbit-anti-rat antibodies (SouthernBiotech, ITK, Uithoorn, The Netherlands) as the second step and alkaline phosphatase-labeled streptavidin (Dako, Glostrup, Denmark) as the third step. New fuchsin (Dako, Glostrup, Denmark) was used as a chromogen and methyl green for nuclear counterstaining.

Frozen sections were stored at -20°C until use. After thawing, slides were incubated in acetone for 10 min. Subsequently, sections were incubated with specific antibodies for CD3, CD4 and CD8-positive T-cells (antibodies from BD Pharmingen). Detection of CD4 and CD8 antibodies was performed using biotin-labeled goat-anti-rat antibodies as the second step. Anti mouse-CD3 polyclonal serum (from hamster) was detected by polyclonal biotin-labeled mouse-anti-hamster antibodies (BD Pharmingen) and the third step was streptavidin-peroxidase complex (Dako, Glostrup, Denmark). Amino-ethyl carbazole (AEC) (Sigma Aldrich, Zwijndrecht, The Netherlands) was used as chromogen, and hematoxylin was used for nuclear counterstaining. All incubation steps were carried out at room temperature. PBS/1% BSA or irrelevant isotype specific antibodies were used as negative controls. Mouse spleen was used as positive control.

### Statistical analysis

All data are expressed as the mean ± SD. Multiple linear regression analysis with an interaction term was used to establish the statistical significances of differences in terms of genotype (TKO or WT) and cigarette smoke exposure for each parameter. This method disentangles the separate effects of cigarette smoke and genotype and their interaction. A significant interaction indicates that the effect of the combination is significantly different (larger or smaller) than the addition of the separate effects of smoke exposure and the genotype. When the interaction term was significant, the regression coefficients of the separate effects of smoking and of genotype were taken from this model. In cases of no significant interaction, the interaction term was removed from the analysis and the coefficients were taken from the model with only smoking and genotype. The normal distribution was tested with a Kolmogorov-Smirnov test and data were logarithmically transformed when needed to normalize distributions. A value of *P *< 0.05 was considered significant. Statistical analyses were performed with SPSS 10 (SPSS Inc, Chicago, IL).

## Results

Two sham control mice of the WT group died of unknown cause in the last month before ending the experiment. Autopsy did not reveal specific pathology, in particular no indications of infection. Malignancies were not observed in lungs of either WT or TKO mice.

### Cytokines and chemokines

**Figure 1:**
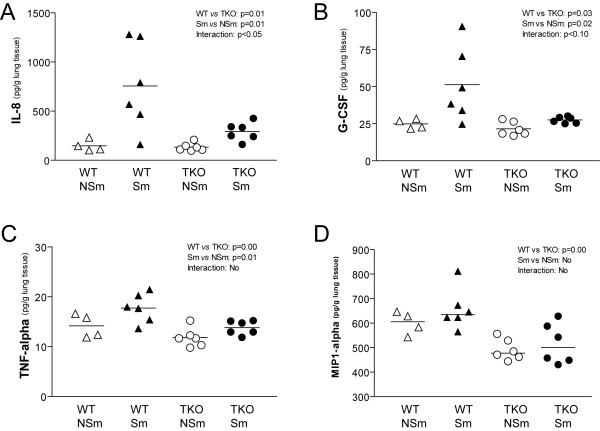
Cytokine and chemokine levels in lung homogenates after 6 months smoke or air exposure. **(A) **IL-8 levels were elevated by cigarette smoke in total group (WT and TKO) of smokers (*P *= 0.01). IL-8 levels are higher in the total group (Sm and NSm) of WT mice compared to TKO mice (*P *= 0.01). The smoke-induced upregulation of IL-8 in lungs of WT was higher compared to TKO mice (interaction, *P *< 0.05). **(B) **Granulocyte-colony stimulating factor (G-CSF) levels were elevated by cigarette smoke in total group (WT and TKO) of smokers (*P *= 0.02). G-CSF levels are higher in the total group (Sm and NSm) of WT mice compared to TKO mice (*P *= 0.03). There was a trend to higher smoke-induced upregulation of G-CSF in lungs of WT compared to TKO mice (interaction, *P *< 0.10). **(C) **TNFα levels were elevated by cigarette smoke in total group (WT and TKO) of smokers (*P *= 0.01) and TNFα levels were higher in the total group (Sm and NSm) of WT mice compared to TKO mice (*P *= 0.00). There was no difference in upregulation of smoke-induced TNFα in lungs of WT versus TKO mice (no interaction). **(D) **MIP1α levels in total group of smokers compared to non-smokers was not significant, but levels are higher in the total group (Sm and NSm) of WT mice compared to TKO mice (*P *= 0.00). There was no difference in smoke-induced upregulation of MIP1α in lungs of WT versus TKO mice (no interaction). *WT: Wild-type; TKO: Mrp1/Mdr1a/1b triple knock-out; Sm: smoker; NSm: non-smoker*.

Levels of IL-7, IL-8, IL-12p70, IL-17, TNFα, G-CSF, GM-CSF and MIP1α in whole lung homogenates were significantly lower in TKO mice than WT mice independent of smoke exposure (Table 1). After 6 months smoke exposure, IL-1α, IL-6, IL-8, IL-13, IL-17, TNFα, G-CSF, GM-CSF and MCP-1 in lungs of mice were elevated in TKO and WT mice (Table 1). The results of IL-8, G-CSF, TNFα, and MIP1α measurements are depicted in Figure 1A–D. The increase of IL-8 in response to smoke was less in TKO mice compared to WT mice (*P *= 0.047), with a similar trend for G-CSF production (*P *= 0.096) (Figure 1 and Table 1, smoke × genotype interaction term).

**Table 1:** Cytokines and chemokines in lung homogenates.

Cytokines/Chemokines	Values in pg/g lung homogenate (mean (SD))				*P*-values		
	
	WT-NSm	WT-Sm	TKO-NSm	TKO-Sm	Smoke × Genotype interaction	Smoke effect	Genotype effect
IL-1α	118.4 (38.1)	195.3 (42.8)	120.8 (22.0)	158.6 (40.8)	NS	**0.00 **↑	NS
IL-1β	54.0 (10.6)	61.5 (12.8)	50.4 (6.6)	51.7 (6.6)	NS	NS	NS
IL-2	87.9 (35.9)	80.3 (20.3)	70.8 (13.3)	80.3 (23.5)	NS	NS	NS
IL-4	4.5 (0.8)	5.0 (0.6)	4.1 (0.6)	4.7 (0.7)	NS	NS	NS
IL-5	23.6 (3.8)	24.4 (7.1)	22.7 (3.6)	23.9 (6.6)	NS	NS	NS
IL-6	45.4 (11.6)	111.7 (58.0)	47.5 (6.0)	76.0 (17.7)	NS	**0.01 **↑	NS
IL-7	192.1 (26.4)	196.2 (28.0)	162.0 (9.8)	183.2 (24.4)	NS	NS	**0.05 **↓
IL-8 (KC)	147.1 (58.2)	755.2 (448.0)	133.1 (41.1)	291.8 (93.7)	**0.05 **↓	**0.01 **↑	**0.01 **↓
IL-9	136.3 (38.4)	123.6 (21.0)	111.3 (25.1)	107.9 (14.5)	NS	NS	NS
IL-10	359.6 (124.4)	359.4 (103.9)	384.4 (186.4)	366.9 (48.4)	NS	NS	NS
IL-12p70	213.9 (30.7)	213.6 (30.6)	173.9 (15.5)	198.9 (21.0)	NS	NS	**0.03 **↓
IL-13	40.3 (5.3)	49.8 (13.8)	36.8 (2.7)	45.9 (7.5)	NS	**0.02 **↑	NS
IL-15	99.0 (21.7)	108.0 (15.8)	84.9 (7.4)	98.2 (11.9)	NS	NS	NS
IL-17	20.4 (2.5)	34.2 (16.3)	17.6 (1.4)	20.8 (1.8)	NS	**0.05 **↑	**0.04 **↓
TNFα	14.2 (2.4)	17.7 (2.9)	11.8 (1.9)	13.8 (1.4)	NS	**0.01 **↑	**0.00 **↓
IFNγ	80.5 (24.4)	73.11 (14.6)	80.0 (25.4)	78.3 (12.6)	NS	NS	NS
G-CSF	24.9 (3.2)	51.3 (24.8)	21.5 (4.7)	27.5 (2.1)	**0.10 **↓	**0.02 **↑	**0.03 **↓
GM-CSF	36.2 (5.7)	40.5 (5.4)	30.1 (1.4)	35.4 (5.7)	NS	**0.03 **↑	**0.01 **↓
IP-10	297.8 (116.5)	431.9 (304.5)	232.9 (108.4)	220.7 (74.1)	NS	NS	NS
MCP-1	38.0 (2.4)	46.9 (7.2)	29.1 (2.0)	48.2 (24.2)	NS	**0.02 **↑	NS
MIP1α	600.1 (46.4)	657.3 (83.7)	491.3 (42.9)	516.0 (82.1)	NS	NS	**0.00 **↓
RANTES	37.4 (8.7)	50.3 (38.0)	57.1 (43.9)	33.9 (11.4)	NS	NS	NS

WT wild-type; TKO triple knock-out mice; Sm smokers; NSm non-smokers; SD standard deviation; NS not significant. See text for further details.

### Evaluation of inflammatory cells and emphysema

**Figure 2:**
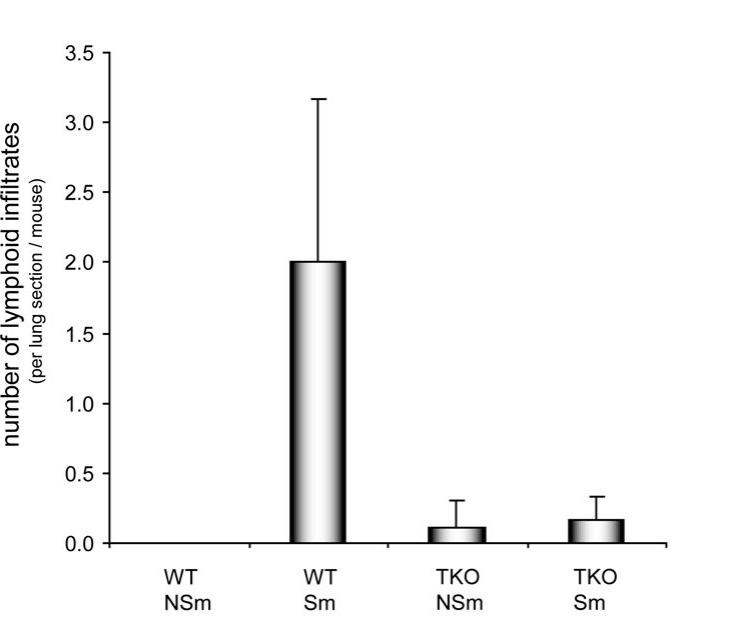
Number of lymphoid infiltrates in paraffin sections of lungs of WT mice and *Mrp1/Mdr1a/1b *TKO mice that were exposed to cigarette smoke or air for 6 months. The number of smoke-induced lymphoid infiltrates in lungs of WT was higher compared to TKO mice (interaction,* P *= 0.02). *WT: Wild-type; TKO: Mrp1/Mdr1a/1b triple knock-out; Sm: smoker; NSm: non-smoker*.

**Figure 3:**
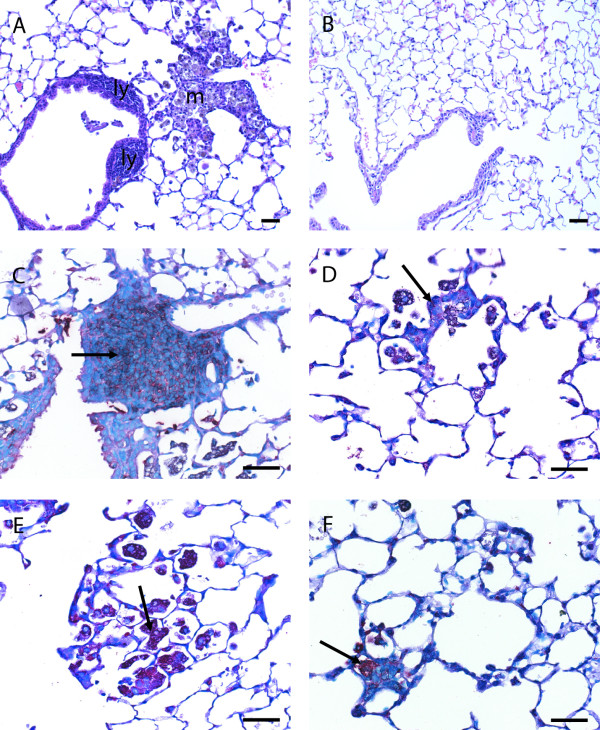
Histological pictures of paraffin sections of lungs of WT mice **(A, C and E) **and *Mrp1/Mdr1a/1b *TKO mice **(B, D, and F) **that were exposed to cigarette smoke for 6 months. **(A, B) **H&E staining of lungs of WT and TKO mice. In WT mice there were markedly more inflammatory infiltrates than in lungs of TKO mice. Two types of infiltrates could be distinguished in paraffin sections, infiltrates mainly consisting of pigmented (smoke particles positive) macrophages (m) and infiltrates mainly consisting of lymphoid (ly) cells. **(C, D) **Lymphoid infiltrates mainly consisted of B-cells (B220 antibody) which were far more present in lungs of WT mice compared to TKO mice, see arrows. **(E, F) **Pigmented macrophages stained positive with specific antibodies (Mac-3), and were far more present in lungs of WT mice compared to TKO mice, see arrows. *Scale bar = 25 μM*.

There were significantly lower numbers of lymphoid inflammatory cells in the paraffin lung sections of smoke-exposed TKO mice as compared to WT mice (*P *= 0.02) (Figure 2). Lymphoid infiltrates as well as pigmented (smoke particles positive) macrophages could be distinguished. Representative pictures are shown in Figure 3. Almost no infiltrates were detected in the lungs of TKO and WT mice that were not exposed to smoke. The lymphoid infiltrates consisted mainly of B-cells surrounded by CD4 positive and CD8 positive cells and their relative distribution was comparable between WT and TKO smoke-exposed mice. Neutrophils were present in very low numbers in all experimental groups and were not further analyzed.

**Figure 4:**
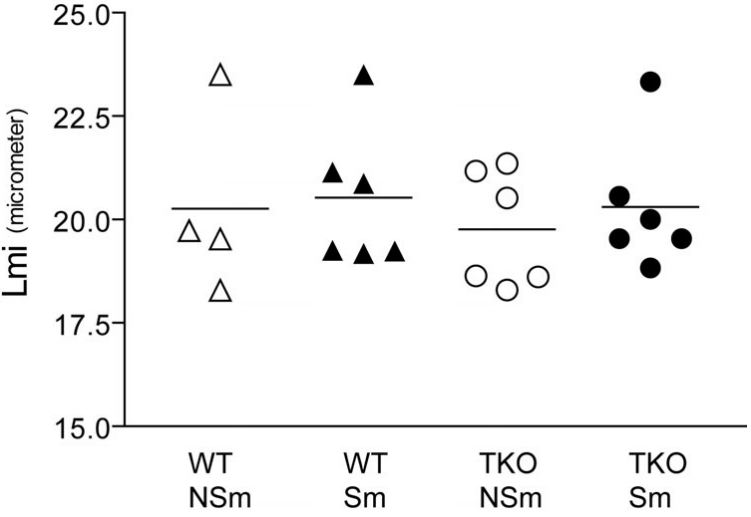
Emphysema measurement with the Lmi method in mice that were exposed to cigarette smoke or air for 6 months. Paraffin sections were stained with H&E and digital images (19 to 32 images per lung) were morphometrically analyzed. No significant differences were measured between the four groups, although a slight increase in airspace size was detected in both WT and TKO mice that were exposed to smoke. *WT: Wild-type; TKO: Mrp1/Mdr1a/1b triple knock-out; Sm: smoker; NSm: non-smoker; Lmi: mean linear intercept*.

Mean Lmi values ranged from 18.3 to 23.5 μm (Figure 4). There was a slight increase of airspace size after 6 months smoke exposure in both TKO and WT mice. However, no significant differences in Lmi values were measured between the four groups. The body weight of the mice was not significantly different between the groups before and after smoke exposure.

## Discussion

Our data show that the inflammatory response to cigarette smoke exposure in lungs of *Mrp1/Mdr1a/1b *TKO mice is reduced as compared to WT mice. This is illustrated by lower numbers of inflammatory cells in lung sections as well as by lower levels of IL-8 and G-CSF in total lung extracts. This is the first study that reports lower smoke-induced pulmonary inflammation in relation to absence of ABC transporters. In addition, the expression levels of several cytokines and chemokines were also lower in lungs of TKO mice independent of smoke exposure.

A possible explanation of the lower inflammatory response in TKO mice is an impaired transport of the pro-inflammatory mediator LTC4, an important physiological high-affinity substrate for MRP1 [10]. In a study with inoculation of *Streptococcus pneumoniae, Mrp1 *(-/-) mice displayed a better survival compared to WT mice. This was accompanied by a lower LTC4 concentration but a higher LTB4 level in bronchoalveolar lavage fluid [17]. In contrast, the outgrowth of *Mycobacterium tuberculosis *was enhanced in *Mrp1 *(-/-) mice [18]. Other interesting observations are that it has been shown that MRP1 regulates the migration of dendritic cells by transporting LTC4, which acts as a chemoattractant for dendritic cells to lymph nodes [19]. The differentiation of dendritic cells is also dependent on MRP1 function [20]. Furthermore, in *Mdr1a *(-/-) mice, migration of dendritic cells to draining lymph nodes is impaired [21]. The above-mentioned observations on dendritic cells may contribute to the decreased inflammatory response following smoke exposure that we measured in lungs of TKO mice.

IL-8 levels were markedly decreased upon smoke exposure in lungs of TKO mice. IL-8 is induced by smoking and elevated in lungs of COPD patients [22]. It is mainly excreted by neutrophils, alveolar macrophages and epithelial cells and a chemoattractant for neutrophils. This is consistent with the decrease in number of macrophages that we observed in lung tissue of TKO mice after smoke exposure. Neutrophils were present in low numbers in both WT and TKO mice, thus, differences in neutrophil recruitment by IL-8 to the lungs could not be detected. Intriguingly, IL-8 is known to be involved in tissue repair reactions [23] and both the IL-8 and G-CSF response tended to be lower in TKO mice. G-CSF has been reported to promote lung regeneration as well, supposedly by mobilization of bone marrow derived cells to alveolar tissue [24]. However, the precise role of IL-8 and G-CSF in COPD development and/or prevention remains to be investigated. The decreased excretion of these two cytokines might result in a delayed lung regeneration mechanism. This question cannot be answered in this study, as 6 months smoking was not sufficient to induce emphysema in these WT and TKO mice (see discussion below).

The main question is whether the reduced inflammatory response in TKO mice could be related to the development of COPD. In a longitudinal study, we recently reported that COPD patients who stopped smoking had an increase in inflammatory cells in induced sputum and we hypothesized that this might actually be beneficial by augmenting the defense capacity [25]. Therefore, the decrease in inflammatory response in TKO mice in our current study could be detrimental instead of beneficial, but we currently have no further data to support this hypothesis.

We have previously detected lower MRP1 expression in bronchial epithelium of COPD patients compared to healthy matched controls who were all ex-smokers [12]. These results support our original hypothesis that lower expression is associated with COPD development and hence we anticipated more detrimental effects of cigarette smoke in *Mrp1 *knock-out or *Mrp1/Mdr1a/1b *TKO mice. Further support for this hypothesis comes from studies with NRF2 (nuclear factor-E2 p45-related factor). NRF2, a transcription factor for many genes that play a role in antioxidant defense and detoxification processes, was recently also identified as a transcription factor for MRP1 [26]. Interestingly, the onset of cigarette smoke-induced emphysema was earlier and the extent of emphysema was more severe in *Nrf2 *(-/-) mice than in wild-type mice [27]. The number of inflammatory cells (mainly macrophages) in bronchoalveolar lavage fluid and lung tissue was higher in *Nrf2 *(-/-) mice. The absence of only the two proteins Mrp1 and P-gp in our knock-out model compared to the absence of the transcription factor NRF2 that regulates many genes involved in oxidative defense, may explain differences in outcome of this study and ours.

The smoke induced pulmonary infiltrates consisted of B-cells surrounded by T-cells and macrophages. Hogg *et al*. observed such follicles in the small airways of humans with COPD [28] and we described these B-cell follicles in our smoking mouse model of emphysema and in lung parenchyma of patients with COPD [15]. We hypothesized that these B-cells secrete antibodies directed against extracellular matrix proteins or tobacco smoke components but the source of the antigen(s) remains to be elucidated. D'hulst and colleagues have recently shown smoke-induced emphysema in SCID mice [29]. They suggested that functional B- and T-cells are not required to induce emphysema but, as they also point out, this does not exclude the possibility that the lymphoid follicles could contribute to loss of alveolar tissue and the decline in lung function in COPD patients.

We did not observe emphysema in both WT and TKO mice after 6 months of smoke exposure. With the same smoke exposure protocol, we have successfully induced emphysema in C57BL/6J mice [15]. Possibly, FVB mice are more resistant to smoke-induced emphysema than C57BL/6J mice, as differences in vulnerability are known to occur between mouse strains [30,31]. To measure differences in alveolar destruction or lung cancer, smoke exposure for a longer period of time or higher smoke doses may be required. We used the Lmi method for evaluation of emphysema development that measures air space enlargement only. Lmi is an artificial measurement of frequency of presence of structures like alveolar walls. Several other methods can be used to measure features of emphysema including "destructive index" and "internal surface area". These methods capture other aspects of emphysema but have high correlations with Lmi. However, they are considerably more time consuming. Since the results on Lmi were so clearly negative, we chose not to invest in an additional method that most probably would not provide different information; we are unaware of studies with significant differences in one method, that did not even give a trend in another method of emphysema measurement. 

We questioned whether the lack of *Mrp1/Mdr1a/1b *would be compensated for by induction of expression of other MDR proteins of the ATP-binding cassette family i.e. Mrp2, 3, 4, 5, 6, 9 and breast cancer resistance protein (Bcrp). The immunohistochemical expression of these transporters was low or absent, except for Mrp3 which was expressed at the apical side of bronchial eptihelium of FVB mice, however, this may be due to an aberrant staining of this antibody (M3II-2) as other antibodies for Mrp3 were negative in human and mice lung tissue [4]. We observed no differences in expression of all these transporters in *Mrp1/Mdr1a/1b *triple knockout mice compared to wild-type mice, nor did we observe effects of smoking on expression of Mrp1 and P-gp in WT mice or on all other analyzed transporter proteins in lungs of both WT and TKO mice (data not shown). These results indicate that indeed Mrp1 and P-gp are the most important transporters present in the lung and that cigarette smoke exposure did not up- or downregulate expression of MDR proteins.

## Conclusion

The pulmonary inflammatory response to inhalation of cigarette smoke is reduced when *Mrp1 *and both genes for *P-gp *are nonfunctional. This includes in particular a poor ability for smoke-induced IL-8 (and G-CSF) production in *Mrp1/Mdr1a/1b *TKO mice compared to WT mice and altogether, this leads to almost complete absence of inflammatory cells in response to cigarette smoke. An impaired function of MRP1 and/or P-gp may result in insufficient clearance of noxious matter as well. Further research should clarify whether there is a relation between a reduced inflammatory response and impaired tissue repair and thus to an increased risk of developing COPD.

## Competing interests

The author(s) declare that they have no competing interests.

## Authors' contributions

MD drafted the manuscript, processed the lungs, performed Lmi scores and immunohistochemistry and subsequent evalution, and carried out statistical analysis. WT helped with evalution of histology. HT carried out Lmi scores. BS participated in setting up the model for emphysema induction in mice. WT, RS, DP, EV and HK participated in the design of the study and helped with writing the manuscript. All authors read and approved the final manuscript.
